# Genetic Determinants of Long-Term Changes in Blood Lipid Concentrations: 10-Year Follow-Up of the GLACIER Study

**DOI:** 10.1371/journal.pgen.1004388

**Published:** 2014-06-12

**Authors:** Tibor V. Varga, Emily Sonestedt, Dmitry Shungin, Robert W. Koivula, Göran Hallmans, Stefan A. Escher, Inês Barroso, Peter Nilsson, Olle Melander, Marju Orho-Melander, Frida Renström, Paul W. Franks

**Affiliations:** 1 Department of Clinical Sciences, Genetic and Molecular Epidemiology Unit, Lund University, Skåne University Hospital Malmö, Malmö, Sweden; 2 Department of Clinical Sciences, Diabetes and Cardiovascular Disease - Genetic Epidemiology, Skåne University Hospital, Malmö, Sweden; 3 Department of Odontology, Umeå University, Umeå, Sweden; 4 Department of Public Health & Clinical Medicine, Umeå University, Umeå, Sweden; 5 Department of Biobank Research, Umeå University, Umeå, Sweden; 6 NIHR Cambridge Biomedical Research Centre, Institute of Metabolic Science, Addenbrooke's Hospital, Cambridge, United Kingdom; 7 University of Cambridge, Metabolic Research Laboratories Institute of Metabolic Science, Addenbrooke's Hospital, Cambridge, United Kingdom; 8 Wellcome Trust Sanger Institute, Wellcome Trust Genome Campus, Hinxton, Cambridge, United Kingdom; 9 Department of Clinical Sciences, Lund University, Skåne University Hospital, Malmö, Sweden; 10 Department of Clinical Sciences, Hypertension and Cardiovascular Diseases, Skåne University Hospital, Malmö, Sweden; 11 Department of Nutrition, Harvard School of Public Health, Boston, Massachusetts, United States of America; University of Oxford, United Kingdom

## Abstract

Recent genome-wide meta-analyses identified 157 loci associated with cross-sectional lipid traits. Here we tested whether these loci associate (singly and in trait-specific genetic risk scores [GRS]) with longitudinal changes in total cholesterol (TC) and triglyceride (TG) levels in a population-based prospective cohort from Northern Sweden (the GLACIER Study). We sought replication in a southern Swedish cohort (the MDC Study; N = 2,943). GLACIER Study participants (N = 6,064) were genotyped with the MetaboChip array. Up to 3,495 participants had 10-yr follow-up data available in the GLACIER Study. The TC- and TG-specific GRSs were strongly associated with change in lipid levels (β = 0.02 mmol/l per effect allele per decade follow-up, *P* = 2.0×10^−11^ for TC; β = 0.02 mmol/l per effect allele per decade follow-up, *P* = 5.0×10^−5^ for TG). In individual SNP analysis, one TC locus, *apolipoprotein E* (*APOE*) rs4420638 (β = 0.12 mmol/l per effect allele per decade follow-up, *P* = 2.0×10^−5^), and two TG loci, *tribbles pseudokinase 1* (*TRIB1*) rs2954029 (β = 0.09 mmol/l per effect allele per decade follow-up, *P* = 5.1×10^−4^) and *apolipoprotein A-I* (*APOA1*) rs6589564 (β = 0.31 mmol/l per effect allele per decade follow-up, *P* = 1.4×10^−8^), remained significantly associated with longitudinal changes for the respective traits after correction for multiple testing. An additional 12 loci were nominally associated with TC or TG changes. In replication analyses, the *APOE* rs4420638, *TRIB1* rs2954029, and *APOA1* rs6589564 associations were confirmed (*P*≤0.001). In summary, trait-specific GRSs are robustly associated with 10-yr changes in lipid levels and three individual SNPs were strongly associated with 10-yr changes in lipid levels.

## Author Summary

Although large cross-sectional studies have proven highly successful in identifying gene variants related to lipid levels and other cardiometabolic traits, very few examples of well-designed longitudinal studies exist where associations between genotypes and long-term changes in lipids have been assessed. Here we undertook analyses in the GLACIER Study to determine whether the 157 previously identified lipid-associated genes variants associate with changes in blood lipid levels over 10-yr follow-up. We identified a variant in *APOE* that is robustly associated with total cholesterol change and two variants in *TRIB1* and *APOA1* respectively that are robustly associated with triglyceride change. We replicated these findings in a second Swedish cohort (the MDC Study). The identified genes had previously been associated with cardiovascular traits such as myocardial infarction or coronary heart disease; hence, these novel lipid associations provide additional insight into the pathogenesis of atherosclerotic heart and large vessel disease. By incorporating all 157 established variants into gene scores, we also observed strong associations with 10-yr lipid changes, illustrating the polygenic nature of blood lipid deterioration.

## Introduction

The implementation of genome-wide association studies (GWAS) into large, well-characterized cohort collections has spurred the discovery of hundreds of genetic variants for complex cardiometabolic disorders [1]. Of those variants, many have been for blood lipids, with a total of 164 common single nucleotide polymorphisms (SNPs) identified to date at a genome-wide significance level (*P*≤5×10^−8^) [2], [3]. These findings come from large-scale, cross-sectional meta-analyses with sufficient power to detect variants with very small effect-sizes for the corresponding traits (OR≈1.01). Although demonstrating cross-sectional genetic associations is important (e.g., for elucidating biological pathways), from a clinical perspective, the discovery of genetic variants that predict a worsening of lipid levels over time might be more relevant [4]; to our knowledge, no large prospective cohort study focused on the full spectrum of established lipid loci has yet been performed.

The purpose of this study was to examine the predictive ability of 157 established lipid loci (as defined by 164 SNPs), singly and together (genetic risk score (GRS)), on changes in lipid concentrations over a decade of follow-up. Replication analyses in another Swedish cohort were also performed.

## Results

GLACIER Study participant characteristics are shown in Table 1 (baseline only) and Table 2 (longitudinal subset). The cross-sectional MetaboChip genotype data from the GLACIER Study were combined with many other cohorts in one of the prior lipid meta-analyses [3]. Malmö Diet and Cancer (MDC) Study participant characteristics are shown in Table 3.

**Table 1:** Baseline characteristics of the GLACIER Study participants (N = 5,862).

	Data available, N	Mean (SD)	Median (IQR)	%
Sex (male/female)	5,862	-	-	61/39
Age (years)	5,862	48.4 (8.7)	50 (40, 60)	-
BMI (kg/m^2^)	5,862	25.7 (4.0)	25.2 (22.9, 27.7)	-
TG (mmol/l)*	4,335	-	1.32 (1.0, 1.7)	-
TC (mmol/l)	5,839	6.0 (1.3)	5.9 (5.1, 6.8)	-
HDL-C (mmol/l)	3,096	1.4 (0.4)	1.4 (1.2, 1.6)	-
LDL-C (mmol/l)	3,085	4.3 (1.2)	4.3 (3.5, 5.0)	-
High TG (no/yes)	4,335	-	-	73/27
High TC (no/yes)	5,839	-	-	28/72
Lipid lowering medication (no/yes)	5,862	-	-	99/1

BMI - body mass index; HDL-C - high density lipoprotein cholesterol; IQR - interquartile range; LDL-C - low density lipoprotein cholesterol; SD - standard deviation; TC - total cholesterol; TG - triglyceride.

*Only median is reported for TG, as the trait's distribution is not Gaussian.

**Table 2:** Longitudinal characteristics of the GLACIER Study participants (N = 3,495 for TC; N = 2,211 for TG).

		Baseline		Follow-up	
		Mean (SD)	Median (IQR)	Mean (SD)	Median (IQR)
TG subset	Sex (male/female, %)	62/38			
	Age (years)	45.9 (6.4)	50 (40, 50)	55.8 (6.4)	60 (50, 60)
	TG (mmol/l)*	-	1.3 (1.0, 1.7)	-	1.3 (1.0, 1.8)
TC subset	Sex (male/female, %)	61/39			
	Age (years)	45.3 (6.7)	50 (40, 50)	55.2 (6.7)	60 (50, 60)
	TC (mmol/l)	5.7 (1.2)	5.6 (4.9, 6.4)	5.5 (1.1)	5.5 (4.8, 6.2)

BMI - body mass index; IQR - interquartile range; SD - standard deviation; TC - total cholesterol; TG - triglyceride.

*Only median is reported for TG, as the trait's distribution is not Gaussian.

**Table 3:** Longitudinal characteristics of the MDC Study participants (N = 2,943).

	Baseline		Follow-up	
	Mean (SD) or n (%)	Median (IQR)	Mean (SD) or n (%)	Median (IQR)
Sex (male)	1,148 (39)	-	1,148 (39)	-
Age (years)	56.4 (5.7)	56.1 (9.7)	73.0 (5.6)	73.0 (9.2)
TC (mmol/l)	6.11 (1.06)	6.07 (1.42)	5.59 (0.91)	5.60 (1.20)
TG (mmol/l)*	-	1.10 (0.68)	-	1.00 (0.60)
lnTG (ln mmol/l)	0.125 (0.433)	0.095 (0.60)	0.025 (0.425)	0.000 (0.56)
HDL-C (mmol/l)	1.42 (0.37)	1.38 (0.48)	1.42 (0.44)	1.36 (0.59)
LDL-C (mmol/l)	4.13 (0.96)	4.10 (1.20)	3.67 (0.79)	3.60 (0.91)
BMI (kg/m^2^)	25.3 (3.6)	24.9 (4.6)	26.8 (4.4)	26.2 (5.2)

BMI - body mass index; HDL-C - high density lipoprotein cholesterol; IQR - interquartile range; LDL-C - low density lipoprotein cholesterol; SD - standard deviation; TC - total cholesterol; TG - triglyceride.

*Only median is reported for TG, as the trait's distribution is not Gaussian.

The results for cross-sectional analyses in the GLACIER Study are presented in Text S1. The established SNPs explained 8.8%, 4.9%, 9.1% and 4.8% variance for TC, TG, LDL-C and HDL-C, respectively. The weighted GRS (wGRS) (allele counts multiplied by previously published effect sizes for each SNP) explained 7.0%, 3.9%, 6.9% and 2.6% of the variance in TC, TG, LDL-C and HDL-C, respectively.

### Longitudinal analyses

A statistically significant overall decrease in plasma TC concentrations between the baseline and follow-up visits (mean change = −0.18±1.12 mmol/l; *P*<0.0001), but no change in the TG levels (mean change = 0.02±1 mmol/l; *P* = 0.32), was observed.

In individual SNP analysis, Benjamini-Hochberg false discovery rate (FDR) corrected statistically significant associations were observed for the rs6589564 and ΔTG (β = 0.31 mmol/l per allele per decade follow-up, 95% CI: 0.21, 0.41, SE = 0.05, *P_FDR_* = 6.6×10^−7^), rs2954029 and ΔTG (β = 0.09 mmol/l per allele per decade follow-up, 95% CI: 0.03, 0.15, SE = 0.03, *P_FDR_* = 0.009) and rs4420638 and ΔTC (β = 0.12 mmol/l per allele per decade follow-up, 95% CI: 0.06, 0.18, SE = 0.03, *P_FDR_* = 0.002). One additional SNP (rs2131925) showed nominally significant evidence of association with ΔTC (β = 0.07 mmol/l per allele per decade follow-up, 95% CI: 0.03, 0.11, SE = 0.02, *P* = 0.002, *P_FDR_* = 0.083). Seven and five additional SNPs were also nominally statistically associated with ΔTC and ΔTG, respectively (*P*<0.05), but did not survive multiple-test corrections. Nominally significant SNP associations are shown in Table 4 and all longitudinal SNP associations are reported in Table S1.

**Table 4:** Nominally significant SNPs from the longitudinal models in the GLACIER Study (N = 3,495 for ΔTC; N = 2,211 for ΔTG).

Trait	SNP	Locus	EA	EAF	β (95% CI) (mmol/l)	*P*	*P*_FDR_
ΔTC	rs1800562	*HFE*	G	0,93	0.089 (0.003; 0.176)	0.043	0.36
ΔTC	rs2000999	*HPR*	A	0,23	0.072 (0.021; 0.123)	0.006	0.15
ΔTC	rs2072183	*NPC1L1*	C	0,27	0.066 (0.017; 0.114)	0.008	0.15
ΔTC	rs2131925	*ANGPTL3*	T	0,67	0.072 (0.026; 0.119)	2.2×10^−3^	0.08
ΔTC	rs4299376	*ABCG5/8*	G	0,33	0.050 (0.003; 0.098)	0.038	0.36
ΔTC	rs4420638	*APOE*	G	0,20	0.118 (0.064; 0.173)	2.0×10^−5^	1.5×10^−3^
ΔTC	rs6511720	*LDLR*	G	0,92	0.104 (0.026; 0.182)	0.009	0.13
ΔTC	rs6882076	*TIMD4*	C	0,62	0.050 (0.005; 0.095)	0.029	0.36
ΔTC	rs9411489	*ABO*	T	0,16	0.062 (0.004; 0.120)	0.036	0.38
ΔTG	rs11057408	*ZNF664*	G	0,63	0.058 (0.005; 0.112)	0.033	0.29
ΔTG	rs1260326	*GCKR*	T	0,31	0.059 (0.003; 0.116)	0.040	0.24
ΔTG	rs2131925	*ANGPTL3*	T	0,67	0.064 (0.009; 0.120)	0.023	0.25
ΔTG	rs2954029	*TRIB1*	A	0,54	0.092 (0.041; 0.143)	4.1×10^−4^	8.8×10^−3^
ΔTG	rs2972146	*IRS1*	T	0,60	0.057 (0.004; 0.110)	0.034	0.25
ΔTG	rs442177	*KLHL8*	T	0,54	0.060 (0.008; 0.112)	0.023	0.33
ΔTG	rs6589564	*APOA1*	C	0,06	0.308 (0.202; 0.414)	1.5×10^−8^	6.6×10^−7^

95% CI–95% confidence interval; β - beta coefficient; ΔTC - total cholesterol change; ΔTG - triglyceride change; EA - effect allele; EAF - effect allele frequency; FDR - false discovery rate; SE - standard error; SNP - single nucleotide polymorphism

*P* values are based on linear regression models. SNP associations were tested by fitting the previously associated individual variants (additive model) as the independent variables with lipid trait changes as dependent variables. We adjusted the raw *P* values for multiple-testing using Benjamini-Hochberg's FDR.

**Figure 1:**
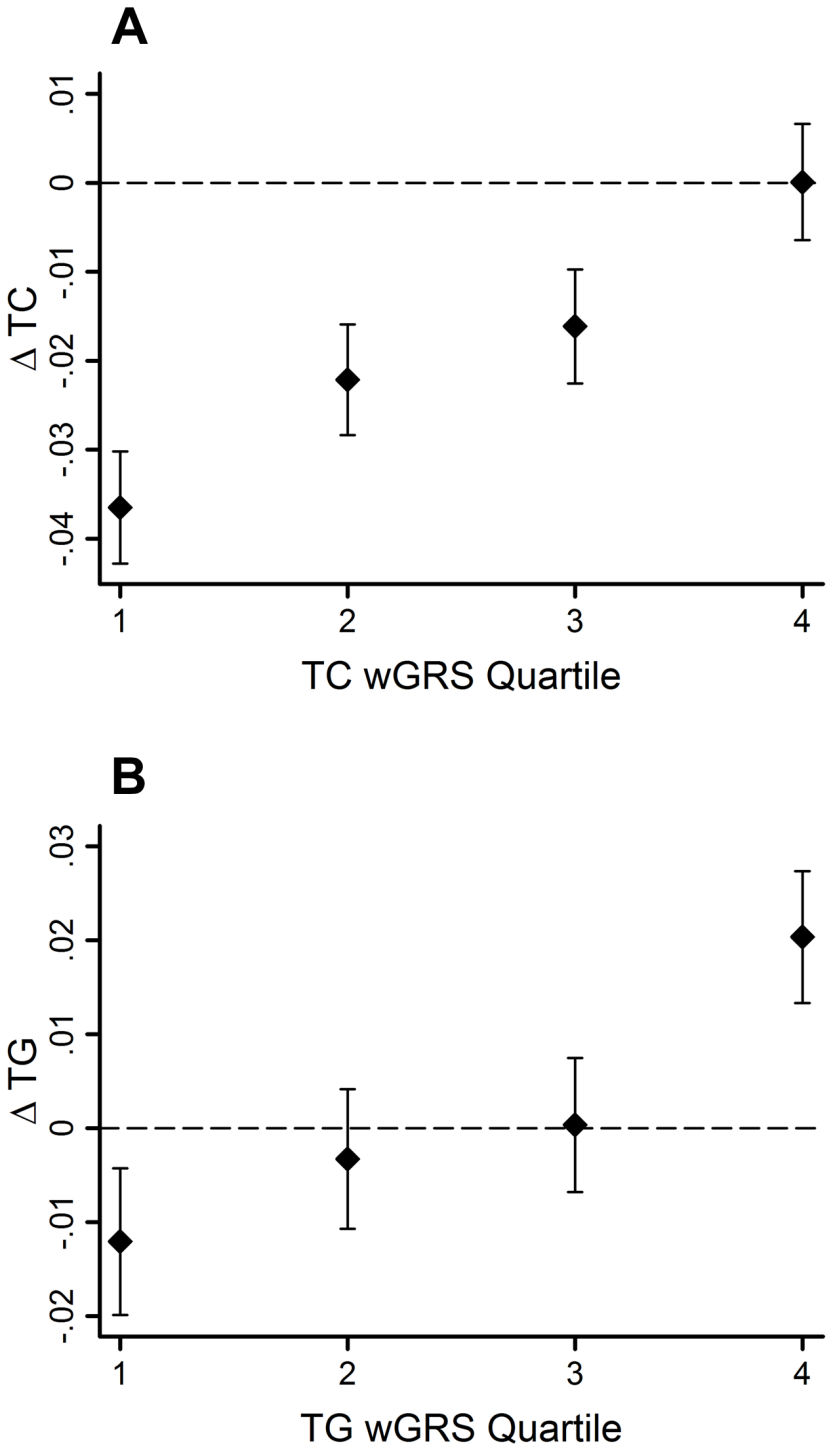
TC and TG level changes (95% CI) over 10-yr follow-up by wGRS quartiles. The TC wGRS was robustly associated with TC changes over follow-up (β = 0.02 mmol/l per allele per follow-up, 95% CI =  0.01, 0.03, SE = 0.003, *P* = 9.8*10^−18^) (**A**). The TG wGRS was robustly associated with TG changes over follow-up (β = 0.03 mmol/l per allele per follow-up, 95% CI =  0.02, 0.04, SE = 0.005, *P* = 6.5*10^−11^) (**B**).

The GRSs were strongly associated with their corresponding trait (β = 0.02 mmol/l per allele per decade follow-up, 95% CI: 0.01, 0.03, SE = 0.003, *P* = 2.0×10^−11^ for ΔTC; β = 0.02 mmol/l per allele per decade follow-up, 95% CI: 0.01, 0.03, SE = 0.005, *P* = 0.0005 for ΔTG). Using the wGRS increased the strength and magnitude of the associations for both traits (β = 0.02 mmol/l per allele per decade follow-up, 95% CI: 0.01, 0.03, SE = 0.003, *P* = 9.8×10^−18^ for ΔTC; β = 0.03 mmol/l per allele per decade follow-up, 95% CI: 0.02, 0.04, SE = 0.005, *P* = 6.5×10^−11^ for ΔTG) (Figure 1A–B). The difference between the highest and lowest quartiles of the wGRS was 0.037 mmol/l for ΔTC, and 0.032 mmol/l for ΔTG.

The variance in lipid changes explained by the wGRS, the baseline lipid measure, sex, age and age^2^ were 33% and 25% for TC and TG, respectively. However, the wGRSs alone explained a small fraction of these proportions (<0.05% for both traits).

**Figure 2:**
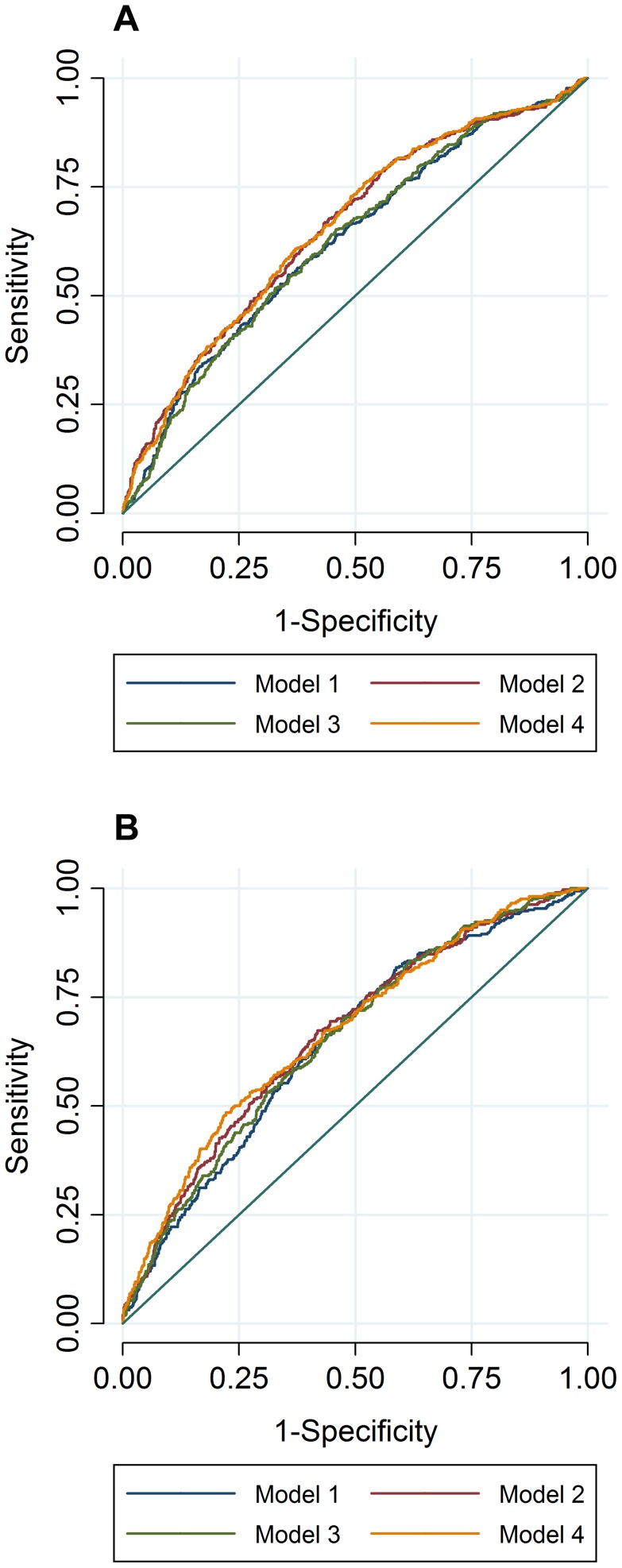
ROC AUC for high TC (A) and high TG (B) at follow-up. In ROC analyses we excluded individuals with hyperlipidemia at baseline and compared the predictive accuracy of four models (age, sex and BMI (Model 1), Model 1 + trait specific wGRS (Model 2), Model 1 + traditional risk factors for hyperlipidemia (Model 3) and M1 + trait specific GRS + traditional risk factors for hyperlipidemia (Model 4)) in relation to hyperlipidemia at follow-up.

To compare the predictive accuracy of traditional risk factors, genetic factors and combined models in relation to hyperlipidemia at follow-up, receiver operating characteristics area under the curve (ROC AUC) analyses were performed. The specificities of the predictive models were above 95%, while the sensitivities of the models were below 20%. The ROC AUC curves are shown in Figure 2A–B, and the pairwise differences and classification statistics in the models for high TC and high TG are shown in Table 5. The lowest ROC AUC values were obtained for the basic models including only age, age^2^, sex and BMI (62% and 65% for high TC and high TG, respectively) and the highest for the combined genetic-lifestyle models (66% and 67% for high TC and high TG, respectively). The difference between these two models was statistically significant for high TC (*P* = 0.011) and approached nominal statistical significance for high TG (*P* = 0.052).

**Table 5:** Pairwise differences between ROC AUC curves and classification statistics in relation to hyperlipidemia in GLACIER (N = 1,257 for TC; N = 1,660 for TG).

		ROC AUC value (%)	Model 1	Model 2	Model 3	Model 4	Sensitivity	Specificity	PPV	NPV	Correctly classified
high TC subset	Model 1	62	-	-	-	-	0.91%	99.63%	57.14%	65.04%	65.00%
	Model 2	65	0.01	-	-	-	17.23%	93.26%	58.02%	67.58%	66.59%
	Model 3	62	0.93	0.02	-	-	2.32%	98.73%	50.00%	64.86%	64.61%
	Model 4	66	0.01	0.48	0.01	-	19.72%	91.61%	56.29%	67.57%	66.17%
high TG subset	Model 1	65	-	-	-	-	3.21%	99.39%	57.89%	79.77%	79.52%
	Model 2	67	0.11	-	-	-	4.66%	99.09%	57.14%	79.96%	79.58%
	Model 3	65	0.27	0.45	-	-	2.47%	99.21%	44.44%	79.86%	79.46%
	Model 4	67	0.05	0.30	0.12	-	4.32%	99.13%	56.00%	80.15%	79.77%

NPV - negative predictive value; PPV - positive predictive value; ROC AUC - receiver operating characteristics area under the curve; TC - total cholesterol; TG - triglyceride.

*P* values are calculated by a chi squared test comparing two ROC AUC curves.

Model 1  =  age,age^2^;sex,BMI; Model 2 =  Model 1+ trait specific GRS; Model 3 =  Model 1+ traditional risk factors (cholesterol intake, trans fat intake, saturated fat intake, carbohydrate intake, alcohol intake, physical activity); Model 4 =  Model 1+ trait specific GRS + traditional risk factors.

### Replication and meta-analysis

As described above, 15 variants (16 associations, as rs2131925 associated with both ΔTC and ΔTG) were nominally associated with change in TG or TC over 10-years follow-up in the GLACIER Study. Results of replication analyses in MDC are presented in Table 6. Associations for five SNPs (rs2131925, rs2954029, rs4420638, rs442177, rs6511720) for ΔTC and six SNPs (rs11057408, rs2072183, rs2131925, rs2954029, rs442177, rs6589564) for ΔTG were nominally statistically (*P*<0.05) significant and directionally consistent with GLACIER results in MDC. Furthermore, four SNPs (rs2954029, rs4420638, rs442177, rs6511720) also associated with ΔLDL-C. None of the SNPs associated with ΔHDL-C in MDC. All three previously associated variants (rs2954029 and rs6589564 in relation to ΔTG and rs4420638 for ΔTC) in GLACIER replicated in MDC.

**Table 6:** Replication of lipid associations in MDC (N = 2,943).

				ΔTC		ΔTG		ΔLDL-C		ΔHDL-C	
SNP	Proxy SNP	EA	EAF	β (95% CI) (mmol/l)	*P*	β (95% CI) (mmol/l)	*P*	β (95% CI) (mmol/l)	*P*	β (95% CI) (mmol/l)	*P*
rs1800562	rs1408272	T	0.95	−0.03 (−0.11; 0.05)	0.50	−0.01 (−0.05; 0.03)	0.48	−0.004 (−0.08; 0.07)	0.91	−0.02 (−0.06; 0.02)	0.25
rs2000999	-	A	0.22	0.02 (−0.02; 0.06)	0.49	0.007 (−0.01; 0.03)	0.54	0.02 (−0.02; 0.06)	0.34	−0.002 (−0.02; 0.02)	0.79
rs2072183	-	C	0.25	0.03 (−0.01; 0.07)	0.27	0.02 (0.00; 0.04)	0.04	0.03 (−0.01; 0.07)	0.12	−0.01 (−0.03; 0.01)	0.13
rs2131925	-	T	0.67	0.04 (0.00; 0.08)	0.05	0.02 (0.00; 0.04)	0.02	0.02 (−0.02; 0.04)	0.20	0.01 (−0.01; 0.03)	0.57
rs4299376	-	G	0.30	−0.01 (−0.03; 0.01)	0.50	−0.01 (−0.03; 0.01)	0.28	−0.02 (−0.06; 0.02)	0.36	0.001 (−0.02; 0.02)	0.99
rs4420638	-	G	0.20	0.07 (0.03; 0.11)	3×10^−3^	−0.004 (−0.02; 0.02)	0.75	0.08 (0.04; 0.12)	4×10^−4^	0.01 (−0.01; 0.03)	0.62
rs6511720	-	G	0.90	0.07 (0.01; 0.13)	0.04	0.004 (−0.04; 0.04)	0.81	0.08 (0.02; 0.14)	7×10^−3^	−0.01 (−0.03; 0.01)	0.42
rs6882076	-	C	0.63	−0.002 (−0.04; 0.04)	0.90	−0.01 (−0.03; 0.01)	0.27	−0.01 (−0.05; 0.03)	0.67	0.01 (−0.01; 0.03)	0.18
rs9411489	rs579459	T	0.77	0.04 (−0.01; 0.08)	0.11	0.004 (−0.02; 0.02)	0.73	0.03 (−0.01; 0.07)	0.21	0.01 (−0.01; 0.03)	0.20
rs11057408	rs4765127	G	0.66	0.001 (−0.04; 0.04)	0.95	0.02 (0.00; 0.04)	0.04	0.01 (−0.03; 0.05)	0.68	−0.01 (−0.03; 0.01)	0.17
rs1260326	-	T	0.36	0.001 (−0.04; 0.04)	0.95	0.02 (−0.00; 0.04)	0.08	0.01 (−0.03; 0.05)	0.64	−0.01 (−0.03; 0.01)	0.18
rs2954029	-	A	0.52	0.04 (0.00; 0.08)	0.05	0.04 (0.02; 0.06)	1×10^−4^	0.04 (0.00; 0.08)	0.03	−0.01 (−0.03; 0.01)	0.55
rs2972146	-	T	0.63	−0.01 (−0.05; 0.03)	0.47	0.01 (−0.01; 0.03)	0.53	−0.004 (−0.04; 0.04)	0.83	−0.01 (−0.03; 0.01)	0.32
rs442177	-	T	0.57	0.04 (0.00; 0.08)	0.04	0.03 (0.01; 0.05)	1×10^−3^	0.03 (−0.01; 0.07)	0.05	−0.003 (−0.02; 0.02)	0.73
rs6589564	rs9326246	C	0.06	0.03 (−0.05; 0.11)	0.41	0.07 (0.03; 0.11)	2×10^−4^	0.03 (−0.05; 0.11)	0.34	−0.02 (−0.06; 0.02)	0.29

95% CI–95% confidence interval; β - beta coefficient; ΔHDL-C - high density lipoprotein cholesterol change; ΔLDL-C - low density lipoprotein cholesterol change; ΔTC - total cholesterol change; ΔTG - triglyceride change; EA - effect allele; EAF - effect allele frequency; SE - standard error; SNP - single nucleotide polymorphism

*P* values for lipid changes are based on linear regression models, marginal effects were tested by fitting the previously statistically nominally significantly associated single variants (additive model) as the independent variables with lipid trait changes as dependent variables.

Meta-analysis results for the 15 longitudinally associated variants are shown in Table S2. Three ΔTC associated variants and six ΔTG associated variants had statistically significant pooled effects (*P*<0.05).

## Discussion

This study extends work reported in two recent large-scale cross-sectional GWAS meta-analyses for lipid loci [2], [3] by examining these variants in the setting of a prospective cohort study (10-yrs follow-up). The trait-specific GRSs were strongly associated with their corresponding lipid traits in both cross-sectional and longitudinal models. Three previously associated variants yielded statistically significant main effects in the longitudinal analyses, namely the *APOA1* rs6589564 and ΔTG (*P_FDR_* = 7.3×10^−7^), the *TRIB1* rs2954029 and ΔTG (*P_FDR_* = 0.013) and the *APOE* rs4420638 and ΔTC (*P_FDR_* = 0.002). We used rs6589564 as the best available proxy in our panel for the *APOA1* rs964184 variant (distance = 24.8 kb; r^2^ = 0.688; D' = 1) [5]. Tentative evidence for association was observed for *angiopoietin-like 3* (*ANGPTL3*) rs2131925 and ΔTC (*P* = 0.002, *P_FDR_* = 0.083), which given the high prior for association, likely reflects an additional locus that influences changes in lipid levels. These statistically significant associations in GLACIER were successfully replicated in MDC (*P* = 4.0×10^−5^ for *APOA1* rs6589564 and ΔTG; *P* = 5.0×10^−5^ for *TRIB1* rs2954029 and ΔTG; *P* = 0.01 for *APOE* rs4420638 and ΔTC; *P* = 0.03 for *ANGPTL3* rs2131925 and ΔTC) and 9 of the 16 nominally significant associations in GLACIER remained significant after meta-analyzing the two cohorts. In ROC analyses, the combined genetic-lifestyle model had higher predictive ability than other models for both traits, but after Bonferroni correction of ROC AUC comparative *P* values, this difference was not statistically significant.

Two large, recent cross-sectional meta-analyses identified a total of 164 new variants associated with blood lipid levels [2], [3]. Whilst these studies highlight numerous, previously unknown biologic pathways underlying dyslipidemia, they have focused exclusively on cross-sectional data, which may not be informative of the genetic mechanisms underlying the deterioration of blood lipid profiles. Prospective data is clinically more relevant, as knowledge of loci that predict change in lipids over time may provide information for clinical translation and risk prediction [4]; however, the extent to which clinical translation could be realized depends on achieving a high level of predictive accuracy using genetic risk algorithms, which at present is not the case for common cardiometabolic diseases [6]. A small number of prospective genetic association studies for lipid loci have been reported [7]–[10], but these studies have focused on only a handful of the 157 established lipid-loci. In the present study, we show that the ability of these established lipid loci to predict incident dyslipidemia is low in these Swedish populations; adding the wGRS to the risk prediction model incorporating the conventional risk factors for hyperlipidemia (comparing Model 3 and Model 4 (shown in Table 5)) increased the AUC values by 4% and 2% for high TC and high TG, respectively. This is comparable to the 3% AUC difference for incident hypercholesterolemia reported by Lu et al., although they used an unweighted GRS of only 12 established TC variants [10].

Teslovich *et al*. reported ∼12% variance explained by the 95 loci discovered in their meta-analysis for TC, TG, LDL-C and HDL-C [2]. The 62 lipid loci recently discovered by Willer *et al*. explain an additional ∼2% of the variance per lipid trait [3]. In Lu *et al*. 's report, 12 candidate SNPs explained 6.9% of the variance in TC, while Sabatti *et al*. attributed 4.8%, 6%, and 6% of the total variance in TG, LDL-C and HDL-C to 4-11 GWAS identified SNPs [10], [11]. In cross-sectional analyses in the GLACIER Study the variances explained by the established SNPs for TC, TG, LDL-C and HDL-C were 8.8%, 4.9%, 9.1% and 4.8%, respectively.

Aulchenko *et al*. used GRSs comprising 7-11 lipid loci; the variances explained by these SNPs were 3.9%, 3.0%, 3.4% and 4.8% for TC, TG, LDL-C and HDL-C, respectively [12]. Lutsey *et al*. evaluated the explained variance by trait specific GRSs, in which they incorporated the 95 loci identified by Teslovich *et al*.; the explained variance for TC, TG, LDL-C and HDL-C were 6.8%, 6.0%, 6.0% and 1.6%, respectively [7]. In the GLACIER Study, the corresponding wGRSs accounted for 7.0%, 3.9%, 6.9% and 2.6% of the trait variances, respectively.

The *TRIB1* locus, which harbors one of the variants (rs2954029) strongly associated with change in TG in our study, encodes a protein with a regulatory effect on mitogen-activated protein kinases (MAPKs) [13]. Studies in mice suggest that *TRIB1* plays a role in the transcription of lipogenic genes in hepatocytes and thereby affects overall apolipoprotein B (ApoB) particle accumulation, alters particle composition and regulates very large density lipoprotein (VLDL), LDL and TG levels [14]. In humans, *TRIB1* variation has been associated with blood lipid levels [2], [3], [15], [16] and increased risk of coronary artery disease [15], [17], ischemic heart disease [18] and myocardial infarction [18]. An *in vitro* study suggested that the protein product of *TRIB1* is in control of vascular smooth muscle cell proliferation and consequently may drive the development of atherosclerosis [19].

We detected a statistically significant association between rs4420638 and TC change. This variant maps to the *APOE*-*APOC1*-*APOC2* cluster on chromosome 19. *APOE* translates to ApoE, which is the main apolipoprotein of the chylomicron, and thus crucial for breaking down TG-rich lipoproteins and essential in maintaining normal plasma cholesterol and TG levels. *APOE* variants have been associated with blood lipid levels [2], [3], [20], familial dyslipoproteinemia [21], polygenic dyslipidemia [22], elevated plasma C-reactive protein levels [23], [24], coronary heart disease [20], [23], and myocardial infarction [15].

We used rs6589564 as a proxy for the *APOA1* rs964184 variant (chromosome 11). Both variants localize to the *APOA1/C3/A4/A5/BUD13* cluster. *APOA1* encodes the major apolipoprotein of plasma HDL particles and plays a central role in lipid metabolism. The rs964184 variant in *APOA1* has been associated with blood lipid levels [2], [3], [22], polygenic dyslipidemia [22], metabolic syndrome [25], coronary heart disease [26], and myocardial infarction [27].

An important strength of this study is the inclusion of replication data. The findings of this study would be enhanced by further investigation of lipoprotein subclasses and the analysis of the effects of lipid lowering interventions in randomized controlled trials.

In conclusion, the trait-specific GRSs were robustly associated with baseline and longitudinal changes in blood lipid concentrations. We detected three novel longitudinal associations in relation to TC and TG changes over a 10-yr follow-up period. As these loci have been previously associated with cardiovascular traits, such as coronary heart disease and myocardial infarction, their associations with lipid changes provide further insight into how these variants contribute to cardiovascular risk.

## Materials and Methods

### Ethics statement

Ethical approval for the GLACIER Study was obtained from the Regional Ethical Review Board in Umeå, Sweden. The Ethics Committee at Lund University approved the MDC study.

### Study participants

The GLACIER Study (N∼19,000) is a prospective, population-based cohort study nested within the Västerbotten Health Survey (VHU) in the northern Swedish county of Västerbotten [28]. Baseline examinations were undertaken from 1985 through 2004. GLACIER participants were invited to attend an examination on their 40th, 50th and 60th birthdays. In a subcohort (N = 5,010), ten-year follow-up data are also available, of whom 3,495 were genotyped (see below). Anthropometric measures (age, sex, height and weight) were collected, and detailed assessments of lifestyle were obtained using a validated questionnaire [28], [29]. All participants provided written informed consent as part of the VHU. The MDC Study constitutes southern Swedish adults participating in a cardiovascular program, with baseline data recorded from 1991 through 1996 [30], [31]. All individuals who were alive and still living in Sweden were invited for follow-up between 2007 and 2012. A total of 3,734 individuals attended follow-up investigation and 2,943 individuals with no history of coronary events had available data for replications analyses.

### Clinical measures

Clinical measures have been described in detail elsewhere [28], [29]. Capillary blood was drawn following an overnight fast. Serum lipid concentrations were measured on fresh capillary plasma with a Reflotron bench-top analyzer (Roche Diagnostics Scandinavia AB). HDL cholesterol was measured after precipitation of the other lipoproteins with sodium phosphowolframate-magnesium chloride. For the ROC AUC analyses, lipid levels were dichotomized (low/high) according to the American Heart Association criteria [32]. At baseline, 5% of the individuals reported not having fasted for at least 8 hours before the blood draw, and information on fasting time was missing in a further 15% of the participants; therefore analyses were adjusted with a variable indicating fasting status, but this did not materially affect the results. In the MDC Study, TC, TG and HDL-C concentrations in the fasting blood samples were measured with a DAX 48 automatic analyzer (Bayer AB, Göteborg, Sweden) using reagents and calibrators from the supplier of the instrument. HDL-C concentrations were determined by the same procedure as used for TC, but after precipitation of LDL-C and very low-density lipoprotein cholesterol (VLDL-C) with dextran–sulphate [33]. The same laboratory methods where applied for analyzing lipid levels at both visits. Direct anthropometry was measured by nurses. LDL-C concentrations were calculated with the Friedewald formula for both studies [34].

### Lipid medications

One percent of participants reported using lipid-lowering medications, which we controlled for in analyses using a constant, as described by Tobin *et al* [35]. There was no information available on the specific type of the lipid lowering agent used by the participants, but at the time of the examinations the most common class of lipid lowering drugs in northern Sweden was statins, used by ∼96% of lipid lowering medication users [36]. Therefore, to correct lipid levels we used the statin constants proposed by Wu *et al.* [37]: HDL-C: −0.059 mmol/l; LDL-C: +1.279 mmol/l, TC: +1.336 mmol/l, TG: +0.207 mmol/l. The MDC Study participants who reported using lipid-lowering–medication at baseline (3%) were excluded from analyses because the type of medication used could not be determined. 28.3% (n = 834) of the MDC Study participants reported using lipid lowering medication at follow-up; of these, 28% used LDL lowering agents (Crestor, Lipitor, Pravachol, Zocord or Ezetrol) and 0.3% used fibrates (Lopid). Their lipid measures were corrected by adding the appropriate constants proposed by Wu *et al* [37].

### Genotyping

DNA was extracted from peripheral white blood cells and genomic DNA samples were diluted to 4 ng/µl as previously described [38], [39]. Samples were genotyped with the MetaboChip (Illumina iSelect) array [40]. The 102 associated SNPs from Teslovich *et al.* [2] and the 62 SNPs from Willer *et al.* [3] were extracted from the MetaboChip. We used proxy SNPs for 18 variants. Proxies for two SNPs (the TG-associated rs2929282 and the HDL-C associated-rs1047891 variants) were unavailable. Detailed information about the index and proxy SNPs are shown in Table S3. The average genotyping success rate was 99.9%. None of the SNPs deviated significantly from Hardy-Weinberg expectations at a study-wise corrected level (*P*<0.0001). Therefore, we conducted our analyses with a total of 162 SNPs (HDL-C  = 73 SNPs; LDL-C  = 58 SNPs; TC  = 75 SNPs; and TG  = 43 SNPs). For the replication effort in MDC, the SNPs analyzed were genotyped using Illumina OmniExpress Exome. SNP and proxy information are reported in Table S4.

### Genetic risk score

The effects of multiple genetic risk loci on blood lipid traits were studied by constructing two different types of GRS for each study participant. The first assumed an equal magnitude of effect for each risk allele and was generated for each participant by summing the number of risk alleles at each of the associated SNP loci for the respective traits. Thus, because these are all biallelic loci, the GRSs had a minimum possible value of 0 and a maximum possible value of 146, 116, 150 and 86 for HDL-C, LDL-C, TC and TG, respectively. To construct the second GRS, we used published effect sizes for each SNP (from the joint meta-analysis by Willer *et al*. [3]) to weight the contribution of each risk allele. The weighted alleles were subsequently summed into a single weighted GRS (wGRS) as previously described [39], [41]. Missing genotypes were imputed by mean imputation as previously described [42]. To illustrate results in figures we used quartiles of the GRSs.

### Statistical methods

Statistical analyses were undertaken using SAS (version 9.3, SAS Institute Inc., NC, USA), STATA (version 12.1, StataCorp LP, TX, USA), *R* (version 2.15.3, The *R* Foundation for Statistical Computing) and PLINK (version 1.07) [43]–[46]. Main effects were estimated with generalized linear models (GLMs) by fitting genotypes (additive model) or the unweighted/weighted GRSs as the independent variable with the corresponding lipid traits as the dependent variable. We used natural logarithmic transformed TG values for cross-sectional individual SNP analyses and adjusted for age, age^2^, sex, fasting time and population substructure (first four principal components) in all our models. In longitudinal analyses, we included the follow-up lipid measure as the dependent variable and adjusted for the respective trait's baseline value.

*follow-up lipid  = α+β_SNP/GRS_+β_baseline lipid_+β_cov_+…+β_cov_+ε*

For the sake of simplicity, when reporting the estimates for the model above, we refer to ΔTC or ΔTG throughout the manuscript. The Benjamini-Hochberg FDR was used to correct for multiple testing [47]; given the prior knowledge of the SNPs in the analyses, we decided to use a less stringent approach then the Bonferroni or the Holm correction. ROC AUCs were computed and compared using STATA. In these analyses we excluded individuals with hyperlipidemia at baseline and compared the predictive accuracy of four models (age, age^2^, sex and BMI (M1), M1+ trait specific wGRS (M2), M1+ traditional risk factors (age, sex, BMI, smoking status, alcohol intake) [10] for hyperlipidemia (M3) and M1+ trait specific wGRS + traditional risk factors for hyperlipidemia (M4)) in relation to hyperlipidemia at follow-up. Random effects meta-analysis was performed using the *metan* module in STATA [48]. Statistical analyses for MDC were done using SPSS (version 20, IBM Corporation). Linear regression was used to obtain effect sizes (β) and 95% confidence intervals (95% CI) by fitting genotypes (additive model) as the independent variables and follow-up lipid measures (TC, lnTG, HDL-C or LDL-C) as dependent variables. Baseline lipid levels, sex, age and age^2^ were used as covariates. For meta-analysis, regression estimates with average annual lipid level changes as outcome measures were used.

## Supporting Information

Table S1 Longitudinal associations of previously associated SNPs in the GLACIER Study.
(XLSX)

Table S2 Meta-analysis of effect estimates from GLACIER and MDC.
(XLSX)

Table S3 Original and proxy SNPs used in cross-sectional and longitudinal models in the GLACIER Study.
(XLSX)

Table S4 Original and proxy SNPs used in longitudinal models in MDC.
(XLSX)

Text S1 Cross-sectional analyses results in GLACIER.
(PDF)
