# Supplementary material for: Genetic Determinants of Long-Term Changes in Blood Lipid Concentrations: 10-Year Follow-Up of the GLACIER Study
Source: PLoS Genet. 2014 Jun 12;10(6):e1004388. doi: 10.1371/journal.pgen.1004388 (PMC4055682; doi:10.1371/journal.pgen.1004388)
Supplement: Text S1 — Cross-sectional analyses results in GLACIER. (PDF) [file pgen.1004388.s005.pdf]

Text S1 – Cross-sectional analyses results in GLACIER

In the cross-sectional analyses, 48% (36/75) of the TC SNPs, 30% (13/43) of the TG SNPs, 33% (19/58) of the LDL-C SNPs and 14% (10/73) of the HDL-C SNPs were significantly associated ($P<0.05$) with their corresponding traits (Appendix Table). 90% (68/75) of the TC SNPs, 93% (40/43) of the TG SNPs, 84% (49/58) of the LDL-C SNPs and 75% (55/73) of the HDL-C SNPs had directionally consistent beta-coefficients as reported previously. [1] The trait-specific GRSs showed associations in a manner consistent with the published literature (β=0.05 mmol/l per allele, 95% CI= 0.04, 0.06, SE=0.003, $P=3.01*10^{-67}$ for TC; β=0.03 mmol/l per allele, 95% CI= 0.02, 0.04, SE=0.003, $P=4.58*10^{-23}$ for TG; β=0.05 mmol/l per allele, 95% CI= 0.04, 0.06, SE=0.005, $P=2.44*10^{-28}$ for LDL-C; β=-0.01 mmol/l per allele, 95% CI= -0.012, -0.008, SE=0.001, $P=1.35*10^{-11}$ for HDL-C). Using the wGRS strengthened the associations (β=0.06 mmol/l per allele, 95% CI= 0.05, 0.07, SE=0.003, $P=2.43*10^{-101}$ for TC; β=0.02 mmol/l per allele, 95% CI= 0.018, 0.022, SE=0.001, $P=4.24*10^{-41}$ for TG; β=0.06 mmol/l per allele, 95% CI= 0.05, 0.07, SE=0.004, $P=1.17*10^{-50}$ for LDL-C; β=-0.01 mmol/l per allele, 95% CI= -0.012, -0.008, SE=0.001, $P=7.06*10^{-22}$ for HDL-C) (Text S1 Figure A-D). The differences between the highest and lowest quartiles of the GRS were 0.65 mmol/l for TC, 0.30 mmol/l for TG, 0.59 mmol/l for LDL-C and -0.14 mmol/l for HDL-C.

References

1. Global Lipids Genetics, C., et al., *Discovery and refinement of loci associated with lipid levels*. Nat Genet, 2013.

Text S1 Table. Cross-sectional associations of previously associated SNPs in the GLACIER Study

Trait	SNP	EA	EAF	β (95% CI) (mmol/l)	*P*
HDL-C	rs10019888	G	0,14	0,005 (-0,025; 0,034)	0,75
HDL-C	rs1042034	T	0,79	-0,023 (-0,048; 0,003)	0,08
HDL-C	rs10773003	G	0,85	-0,006 (-0,034; 0,023)	0,70
HDL-C	rs1084651	G	0,84	0,024 (-0,003; 0,051)	0,08
HDL-C	rs10850443	G	0,49	-0,004 (-0,025; 0,017)	0,70
HDL-C	rs11057408	G	0,64	-0,001 (-0,022; 0,02)	0,92
HDL-C	rs1121980	A	0,43	-0,023 (-0,043; -0,002)	0,03
HDL-C	rs11246602	T	0,87	-0,008 (-0,038; 0,023)	0,62
HDL-C	rs11613352	C	0,71	-0,026 (-0,048; -0,003)	0,02
HDL-C	rs11869286	G	0,31	-0,004 (-0,026; 0,018)	0,71
HDL-C	rs12145743	T	0,71	-0,008 (-0,031; 0,014)	0,48
HDL-C	rs12328675	T	0,90	-0,001 (-0,035; 0,033)	0,97
HDL-C	rs12678919	A	0,92	-0,009 (-0,046; 0,028)	0,63
HDL-C	rs12748152	T	0,10	-0,012 (-0,046; 0,022)	0,48
HDL-C	rs12801636	G	0,75	-0,014 (-0,038; 0,009)	0,23
HDL-C	rs12967135	A	0,25	-0,002 (-0,026; 0,021)	0,86
HDL-C	rs13107325	T	0,03	-0,042 (-0,102; 0,018)	0,17
HDL-C	rs13326165	G	0,80	-0,025 (-0,051; 0,001)	0,06
HDL-C	rs1532085	G	0,56	-0,054 (-0,075; -0,034)	1.8×10^{-7}
HDL-C	rs1689800	G	0,33	-0,03 (-0,052; -0,008)	0,01
HDL-C	rs16942887	G	0,86	-0,008 (-0,037; 0,022)	0,62
HDL-C	rs17145738	C	0,86	0,011 (-0,019; 0,041)	0,48
HDL-C	rs17173637	C	0,14	-0,005 (-0,035; 0,025)	0,76
HDL-C	rs17404153	G	0,90	0,014 (-0,021; 0,048)	0,45
HDL-C	rs174546	T	0,34	0,008 (-0,014; 0,029)	0,48
HDL-C	rs17695224	A	0,23	-0,014 (-0,038; 0,01)	0,26
HDL-C	rs1800961	T	0,05	-0,03 (-0,075; 0,016)	0,20
HDL-C	rs181362	T	0,24	-0,02 (-0,044; 0,004)	0,10
HDL-C	rs1883025	T	0,25	-0,02 (-0,043; 0,004)	0,10
HDL-C	rs1936800	T	0,55	-0,011 (-0,032; 0,009)	0,29
HDL-C	rs2013208	C	0,52	-0,011 (-0,032; 0,009)	0,27
HDL-C	rs2278236	G	0,50	0,002 (-0,019; 0,022)	0,88
HDL-C	rs2290547	A	0,23	-0,013 (-0,037; 0,011)	0,29
HDL-C	rs2293889	T	0,38	-0,009 (-0,03; 0,011)	0,37
HDL-C	rs2602836	G	0,53	0,003 (-0,017; 0,024)	0,76
HDL-C	rs2606736	T	0,62	0,005 (-0,016; 0,026)	0,61
HDL-C	rs2652834	A	0,20	-0,008 (-0,033; 0,017)	0,53
HDL-C	rs2814944	A	0,13	-0,023 (-0,054; 0,007)	0,13
HDL-C	rs2923084	G	0,19	-0,025 (-0,052; 0,002)	0,07
HDL-C	rs2925979	T	0,32	-0,003 (-0,025; 0,019)	0,80
HDL-C	rs2954029	A	0,52	-0,013 (-0,033; 0,008)	0,22

HDL-C	rs2972146	T	0,59	0,002 (-0,019; 0,022)	0,88
HDL-C	rs3136441	T	0,89	-2×10^{-4} (-0,033; 0,033)	0,99
HDL-C	rs3764261	C	0,64	-0,07 (-0,091; -0,049)	1.5×10^{-10}
HDL-C	rs3822072	A	0,46	0,005 (-0,016; 0,025)	0,66
HDL-C	rs386000	G	0,73	-0,006 (-0,029; 0,017)	0,63
HDL-C	rs4129767	G	0,50	-0,021 (-0,041; 0)	0,05
HDL-C	rs4142995	T	0,35	0,003 (-0,018; 0,024)	0,79
HDL-C	rs4148008	G	0,33	0,001 (-0,021; 0,023)	0,92
HDL-C	rs4420638	G	0,21	-0,004 (-0,03; 0,021)	0,74
HDL-C	rs4650994	A	0,52	0,004 (-0,016; 0,024)	0,70
HDL-C	rs4660293	G	0,24	-0,003 (-0,027; 0,022)	0,82
HDL-C	rs4731702	C	0,48	-0,012 (-0,032; 0,009)	0,26
HDL-C	rs4846914	G	0,43	-0,014 (-0,035; 0,007)	0,18
HDL-C	rs4917014	T	0,74	0,005 (-0,018; 0,029)	0,65
HDL-C	rs4983559	A	0,62	-0,018 (-0,039; 0,003)	0,09
HDL-C	rs499974	A	0,18	-0,012 (-0,038; 0,015)	0,39
HDL-C	rs581080	G	0,20	-0,028 (-0,053; -0,002)	0,03
HDL-C	rs6065906	C	0,22	-0,001 (-0,026; 0,023)	0,93
HDL-C	rs634869	T	0,42	-3.8×10^{-4} (-0,021; 0,021)	0,97
HDL-C	rs6450176	A	0,25	-0,025 (-0,049; -0,002)	0,03
HDL-C	rs6589564	C	0,06	-0,025 (-0,068; 0,018)	0,25
HDL-C	rs6805251	C	0,56	-0,011 (-0,032; 0,009)	0,28
HDL-C	rs702485	A	0,52	0,001 (-0,019; 0,022)	0,92
HDL-C	rs7134375	C	0,57	-0,005 (-0,026; 0,015)	0,61
HDL-C	rs7240405	A	0,17	-0,019 (-0,046; 0,008)	0,17
HDL-C	rs731839	G	0,35	0,01 (-0,011; 0,031)	0,37
HDL-C	rs737337	C	0,14	-0,01 (-0,04; 0,02)	0,50
HDL-C	rs7941030	T	0,62	-0,001 (-0,022; 0,02)	0,91
HDL-C	rs838880	T	0,63	-0,025 (-0,046; -0,004)	0,02
HDL-C	rs970548	A	0,69	0,008 (-0,014; 0,03)	0,48
HDL-C	rs998584	A	0,49	-0,001 (-0,021; 0,02)	0,96
HDL-C	rs9987289	A	0,12	-0,048 (-0,08; -0,016)	3.4×10^{-3}
LDL-C	rs10102164	A	0,22	0,027 (-0,042; 0,097)	0,45
LDL-C	rs10401969	T	0,92	0,133 (0,022; 0,243)	0,02
LDL-C	rs10490626	G	0,93	0,14 (0,023; 0,258)	0,02
LDL-C	rs11065987	A	0,61	-0,008 (-0,068; 0,051)	0,79
LDL-C	rs11220462	A	0,17	0,09 (0,012; 0,167)	0,02
LDL-C	rs11563251	T	0,10	-0,032 (-0,131; 0,066)	0,52
LDL-C	rs1169288	C	0,39	0,032 (-0,028; 0,091)	0,30
LDL-C	rs12027135	T	0,58	0,078 (0,02; 0,136)	0,01
LDL-C	rs1250229	C	0,77	0,036 (-0,033; 0,104)	0,30
LDL-C	rs12670798	C	0,28	0,082 (0,018; 0,145)	0,01
LDL-C	rs12748152	T	0,10	0,076 (-0,02; 0,173)	0,12
LDL-C	rs12916	C	0,41	0,08 (0,021; 0,139)	0,01
LDL-C	rs1367117	A	0,40	0,127 (0,069; 0,185)	1.6×10^{-5}
LDL-C	rs1564348	C	0,15	0,043 (-0,041; 0,126)	0,32

LDL-C	rs17404153	G	0,91	0,036 (-0,062; 0,134)	0,47
LDL-C	rs174546	C	0,66	0,068 (0,006; 0,129)	0,03
LDL-C	rs1800562	G	0,93	0,066 (-0,046; 0,178)	0,25
LDL-C	rs1801689	C	0,01	0,412 (0,048; 0,775)	0,03
LDL-C	rs2000999	A	0,23	0,068 (0; 0,136)	0,05
LDL-C	rs2030746	T	0,37	0,006 (-0,054; 0,066)	0,85
LDL-C	rs2072183	C	0,28	0,043 (-0,021; 0,107)	0,19
LDL-C	rs2131925	T	0,69	0,015 (-0,046; 0,077)	0,63
LDL-C	rs2255141	A	0,24	-0,064 (-0,133; 0,005)	0,07
LDL-C	rs2326077	C	0,35	0,031 (-0,03; 0,092)	0,32
LDL-C	rs2328223	C	0,18	0,012 (-0,062; 0,086)	0,76
LDL-C	rs2479409	G	0,36	0,051 (-0,009; 0,11)	0,10
LDL-C	rs2642442	T	0,70	0,047 (-0,018; 0,112)	0,16
LDL-C	rs267733	A	0,82	0,056 (-0,02; 0,133)	0,15
LDL-C	rs2710642	A	0,67	-0,042 (-0,104; 0,02)	0,19
LDL-C	rs2902940	A	0,70	0,011 (-0,052; 0,073)	0,73
LDL-C	rs2954029	A	0,52	0,078 (0,02; 0,135)	0,01
LDL-C	rs314253	T	0,59	0,042 (-0,017; 0,101)	0,16
LDL-C	rs3177928	A	0,12	0,07 (-0,017; 0,158)	0,11
LDL-C	rs364585	G	0,63	-0,021 (-0,08; 0,038)	0,49
LDL-C	rs3757354	C	0,73	0,069 (0,004; 0,133)	0,04
LDL-C	rs3764261	C	0,64	0,043 (-0,017; 0,104)	0,16
LDL-C	rs3780181	A	0,95	0,075 (-0,057; 0,208)	0,26
LDL-C	rs3798236	T	0,69	-0,017 (-0,079; 0,045)	0,59
LDL-C	rs4253772	C	0,92	-0,002 (-0,105; 0,102)	0,98
LDL-C	rs4299376	G	0,29	0,164 (0,1; 0,227)	5×10^{-7}
LDL-C	rs4420638	G	0,21	0,282 (0,21; 0,354)	2.4×10^{-14}
LDL-C	rs4530754	A	0,47	-0,021 (-0,079; 0,038)	0,49
LDL-C	rs4722551	C	0,22	0,046 (-0,024; 0,116)	0,20
LDL-C	rs4942486	T	0,44	0,079 (0,021; 0,137)	0,01
LDL-C	rs558971	A	0,56	0,067 (0,008; 0,125)	0,02
LDL-C	rs5763662	T	0,02	0,151 (-0,046; 0,348)	0,13
LDL-C	rs6065311	C	0,48	0,046 (-0,012; 0,104)	0,12
LDL-C	rs629301	T	0,75	0,113 (0,047; 0,179)	8.2×10^{-4}
LDL-C	rs6504872	T	0,44	0,01 (-0,048; 0,067)	0,74
LDL-C	rs6511720	G	0,93	0,354 (0,242; 0,467)	6.8×10^{-10}
LDL-C	rs6589564	C	0,06	0,088 (-0,034; 0,21)	0,16
LDL-C	rs6831256	A	0,64	0,039 (-0,021; 0,099)	0,20
LDL-C	rs6882076	C	0,63	0,062 (0,003; 0,121)	0,04
LDL-C	rs7640978	C	0,91	-0,016 (-0,118; 0,086)	0,76
LDL-C	rs7832643	T	0,40	0,033 (-0,027; 0,093)	0,28
LDL-C	rs8017377	A	0,47	0,024 (-0,035; 0,082)	0,43
LDL-C	rs9411489	T	0,16	0,084 (0,006; 0,161)	0,03
LDL-C	rs9987289	G	0,88	0,03 (-0,06; 0,121)	0,51
TC	rs10102164	A	0,23	0,029 (-0,022; 0,079)	0,27
TC	rs10128711	C	0,74	-0,004 (-0,052; 0,045)	0,88

TC	rs10401969	T	0,92	0,169 (0,092; 0,247)	1.8×10^{-5}
TC	rs10490626	A	0,07	-0,07 (-0,153; 0,013)	0,10
TC	rs1077514	T	0,88	0,036 (-0,028; 0,101)	0,27
TC	rs10904908	G	0,41	0,025 (-0,018; 0,067)	0,25
TC	rs11065987	A	0,60	-0,001 (-0,045; 0,042)	0,95
TC	rs11220462	A	0,16	0,071 (0,012; 0,129)	0,02
TC	rs11563251	T	0,10	-0,025 (-0,097; 0,047)	0,50
TC	rs11603023	T	0,39	0,018 (-0,025; 0,061)	0,42
TC	rs1169288	C	0,37	0,047 (0,003; 0,092)	0,04
TC	rs11694172	G	0,28	0,022 (-0,026; 0,07)	0,37
TC	rs11709504	T	0,77	0,044 (-0,006; 0,094)	0,09
TC	rs12027135	T	0,58	0,079 (0,035; 0,122)	3.6×10^{-4}
TC	rs1260326	T	0,30	0,054 (0,008; 0,1)	0,02
TC	rs12670798	C	0,28	0,08 (0,033; 0,127)	9×10^{-4}
TC	rs12916	C	0,41	0,08 (0,037; 0,123)	2.9×10^{-4}
TC	rs13315871	G	0,90	0,024 (-0,045; 0,094)	0,50
TC	rs1367117	A	0,39	0,11 (0,067; 0,154)	6×10^{-7}
TC	rs138777	A	0,40	-0,021 (-0,064; 0,022)	0,34
TC	rs1532085	A	0,44	0,079 (0,036; 0,121)	3×10^{-4}
TC	rs1564348	C	0,14	0,065 (0,003; 0,126)	0,04
TC	rs174546	C	0,66	0,045 (-0,001; 0,09)	0,05
TC	rs1800562	G	0,93	0,143 (0,061; 0,226)	6.6×10^{-4}
TC	rs1800961	C	0,94	0,15 (0,058; 0,241)	1.3×10^{-3}
TC	rs1883025	C	0,74	0,08 (0,032; 0,129)	1×10^{-3}
TC	rs1997243	G	0,15	0,04 (-0,02; 0,1)	0,19
TC	rs2000999	A	0,23	0,055 (0,005; 0,105)	0,03
TC	rs2030746	T	0,38	0,019 (-0,024; 0,063)	0,39
TC	rs2072183	C	0,28	0,053 (0,006; 0,1)	0,03
TC	rs2131925	T	0,68	0,072 (0,026; 0,117)	2×10^{-3}
TC	rs2255141	A	0,24	0,025 (-0,025; 0,075)	0,33
TC	rs2277862	C	0,89	0,006 (-0,063; 0,074)	0,87
TC	rs2287623	G	0,37	0,008 (-0,036; 0,052)	0,72
TC	rs2326077	C	0,34	0,063 (0,018; 0,108)	0,01
TC	rs2479409	G	0,36	0,085 (0,041; 0,129)	1.7×10^{-4}
TC	rs2642442	T	0,71	1.4×10^{-5} (-0,047; 0,047)	1,00
TC	rs2737229	A	0,69	0,053 (0,008; 0,099)	0,02
TC	rs2758886	A	0,24	-0,001 (-0,05; 0,048)	0,97
TC	rs2814982	C	0,91	0,104 (0,032; 0,177)	5×10^{-3}
TC	rs2902940	A	0,71	0,005 (-0,041; 0,052)	0,83
TC	rs2954029	A	0,53	0,092 (0,05; 0,134)	1.8×10^{-5}
TC	rs314253	T	0,60	0,082 (0,039; 0,125)	1.8×10^{-4}
TC	rs3177928	A	0,12	0,102 (0,038; 0,166)	1.9×10^{-3}
TC	rs3757354	C	0,73	0,062 (0,015; 0,109)	0,01
TC	rs3764261	A	0,35	0,054 (0,01; 0,098)	0,02
TC	rs3780181	A	0,95	0,043 (-0,055; 0,142)	0,39

TC	rs3798236	T	0,70	0,007 (-0,039; 0,053)	0,77
TC	rs4253772	T	0,08	0,036 (-0,041; 0,112)	0,36
TC	rs4299376	G	0,28	0,149 (0,102; 0,195)	3.9×10^{-10}
TC	rs4420638	G	0,20	0,239 (0,186; 0,292)	9.9×10^{-19}
TC	rs4530754	A	0,48	0,019 (-0,023; 0,062)	0,38
TC	rs4722551	C	0,21	0,007 (-0,045; 0,059)	0,80
TC	rs4883201	A	0,92	0,037 (-0,044; 0,117)	0,37
TC	rs4921914	C	0,21	0,033 (-0,019; 0,084)	0,22
TC	rs492602	G	0,42	0,034 (-0,008; 0,077)	0,11
TC	rs558971	A	0,55	0,057 (0,014; 0,099)	0,01
TC	rs581080	C	0,80	0,029 (-0,024; 0,082)	0,28
TC	rs6065311	C	0,49	0,056 (0,014; 0,098)	0,01
TC	rs629301	T	0,75	0,089 (0,04; 0,138)	3.5×10^{-4}
TC	rs6504872	T	0,44	0,011 (-0,031; 0,053)	0,62
TC	rs6511720	G	0,92	0,247 (0,169; 0,325)	6×10^{-10}
TC	rs6589564	C	0,06	0,201 (0,113; 0,29)	8.6×10^{-6}
TC	rs6603981	T	0,78	0,066 (0,015; 0,117)	0,01
TC	rs6831256	A	0,63	0,028 (-0,016; 0,071)	0,22
TC	rs6882076	C	0,62	0,032 (-0,011; 0,076)	0,14
TC	rs7240405	G	0,84	0,091 (0,034; 0,148)	1.7×10^{-3}
TC	rs7570971	A	0,26	0,037 (-0,012; 0,085)	0,14
TC	rs7640978	C	0,91	0,05 (-0,026; 0,125)	0,20
TC	rs7832643	T	0,39	0,065 (0,021; 0,109)	3.8×10^{-3}
TC	rs7941030	C	0,38	0,029 (-0,015; 0,072)	0,19
TC	rs9376090	T	0,66	0,025 (-0,02; 0,069)	0,28
TC	rs9411489	T	0,16	0,116 (0,06; 0,173)	5.5×10^{-5}
TC	rs970548	A	0,69	-0,015 (-0,06; 0,031)	0,53
TC	rs9987289	G	0,89	0,032 (-0,038; 0,101)	0,37
lnTG	rs10195252	T	0,58	0,005 (-0,012; 0,022)	0,57
lnTG	rs10401969	T	0,92	0,048 (0,016; 0,08)	3.5×10^{-3}
lnTG	rs1042034	T	0,80	0,031 (0,01; 0,052)	3.7×10^{-3}
lnTG	rs10761741	A	0,63	0,014 (-0,003; 0,031)	0,11
lnTG	rs11057408	G	0,63	0,015 (-0,002; 0,032)	0,09
lnTG	rs1121980	G	0,56	-0,014 (-0,031; 0,002)	0,09
lnTG	rs11613352	C	0,71	0,017 (-0,001; 0,035)	0,07
lnTG	rs11776767	C	0,35	0,008 (-0,011; 0,028)	0,41
lnTG	rs1260326	T	0,30	0,054 (0,036; 0,072)	4.5×10^{-9}
lnTG	rs12678919	A	0,92	0,042 (0,011; 0,073)	0,01
lnTG	rs12748152	T	0,10	0,008 (-0,02; 0,035)	0,59
lnTG	rs13238203	C	0,00	-	-
lnTG	rs1532085	A	0,45	0,007 (-0,01; 0,024)	0,41
lnTG	rs17145738	C	0,87	0,032 (0,008; 0,057)	0,01
lnTG	rs174546	T	0,34	0,014 (-0,003; 0,032)	0,11
lnTG	rs1832007	A	0,81	0,015 (-0,006; 0,036)	0,16
lnTG	rs1936800	T	0,55	0,009 (-0,008; 0,026)	0,29

lnTG	rs2068888	G	0,54	0,028 (0,011; 0,045)	$1.1{\times}10^{-3}$
lnTG	rs2131925	T	0,69	0,023 (0,005; 0,041)	0,01
lnTG	rs2247056	C	0,74	0,008 (-0,011; 0,027)	0,41
lnTG	rs2412710	A	0,02	0,037 (-0,019; 0,092)	0,20
lnTG	rs2954029	A	0,53	0,046 (0,03; 0,062)	$3.6{\times}10^{-8}$
lnTG	rs2972146	T	0,59	0,01 (-0,007; 0,027)	0,24
lnTG	rs3198697	C	0,57	0,013 (-0,004; 0,03)	0,13
lnTG	rs3764261	C	0,65	0,015 (-0,002; 0,032)	0,09
lnTG	rs38855	A	0,52	0,014 (-0,002; 0,031)	0,08
lnTG	rs439401	C	0,64	0,027 (0,01; 0,045)	$1.6{\times}10^{-3}$
lnTG	rs442177	T	0,53	0,014 (-0,002; 0,031)	0,09
lnTG	rs4722551	C	0,21	0,003 (-0,017; 0,024)	0,75
lnTG	rs4846914	G	0,44	0,004 (-0,013; 0,021)	0,64
lnTG	rs4889606	A	0,62	-0,008 (-0,026; 0,009)	0,34
lnTG	rs4921914	C	0,21	0,025 (0,005; 0,046)	0,02
lnTG	rs5756931	T	0,58	0,005 (-0,012; 0,022)	0,59
lnTG	rs6065906	C	0,22	0,022 (0,002; 0,041)	0,03
lnTG	rs645040	T	0,82	0,016 (-0,005; 0,037)	0,14
lnTG	rs6589564	C	0,06	0,098 (0,064; 0,133)	$1.7{\times}10^{-8}$
lnTG	rs6831256	G	0,37	0,017 (-0,001; 0,034)	0,06
lnTG	rs6882076	C	0,62	0,003 (-0,014; 0,02)	0,73
lnTG	rs7248104	G	0,59	0,029 (0,012; 0,046)	$8.8{\times}10^{-4}$
lnTG	rs731839	G	0,36	0,012 (-0,006; 0,029)	0,19
lnTG	rs8077889	C	0,17	0,014 (-0,008; 0,035)	0,22
lnTG	rs9686661	T	0,16	0,011 (-0,012; 0,034)	0,36
lnTG	rs998584	A	0,50	0,006 (-0,01; 0,023)	0,46

95% CI - 95% confidence interval; β - beta coefficient; EA - effect allele; EAF - effect allele frequency; HDL-C - high density lipoprotein cholesterol; LDL-C - low density lipoprotein cholesterol; lnTG - natural logarithm of triglyceride; SE - standard error; TC - total cholesterol. *P* values are based on linear regression models, SNP associations were tested by fitting the previously associated individual variants (additive model) as the independent variables with lipid traits as dependent variables. We did not correct for multiple testing in these models given prior evidence of association.

Text S1 Figure A-D

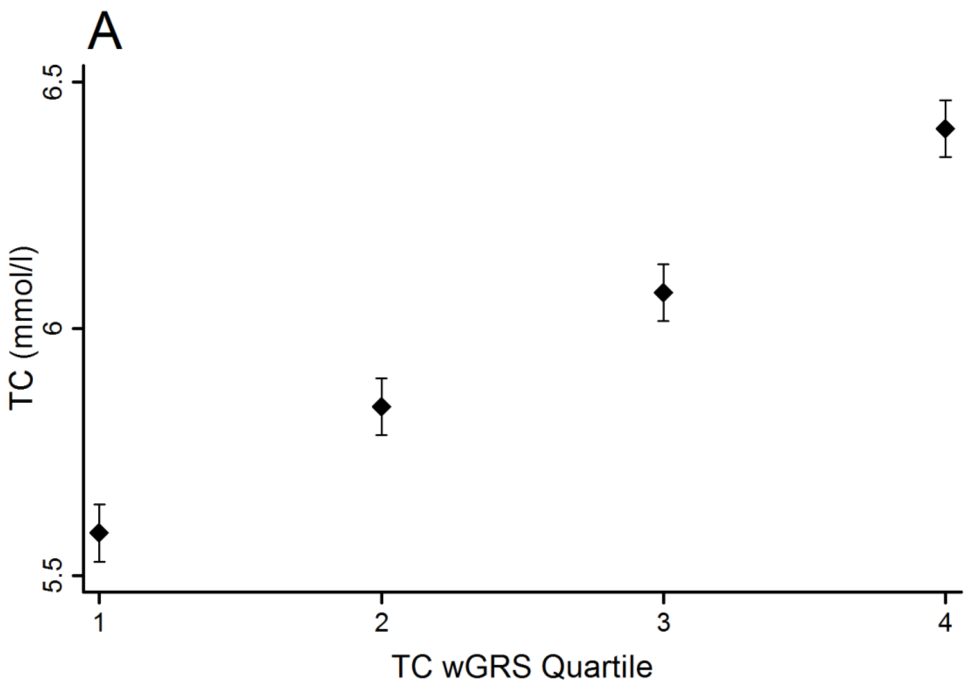

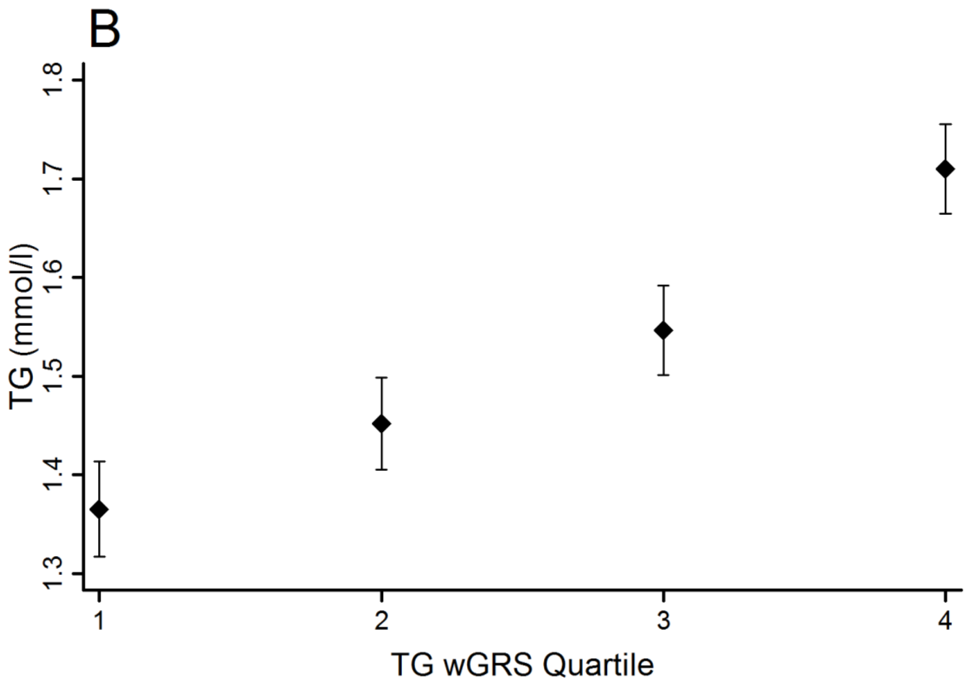

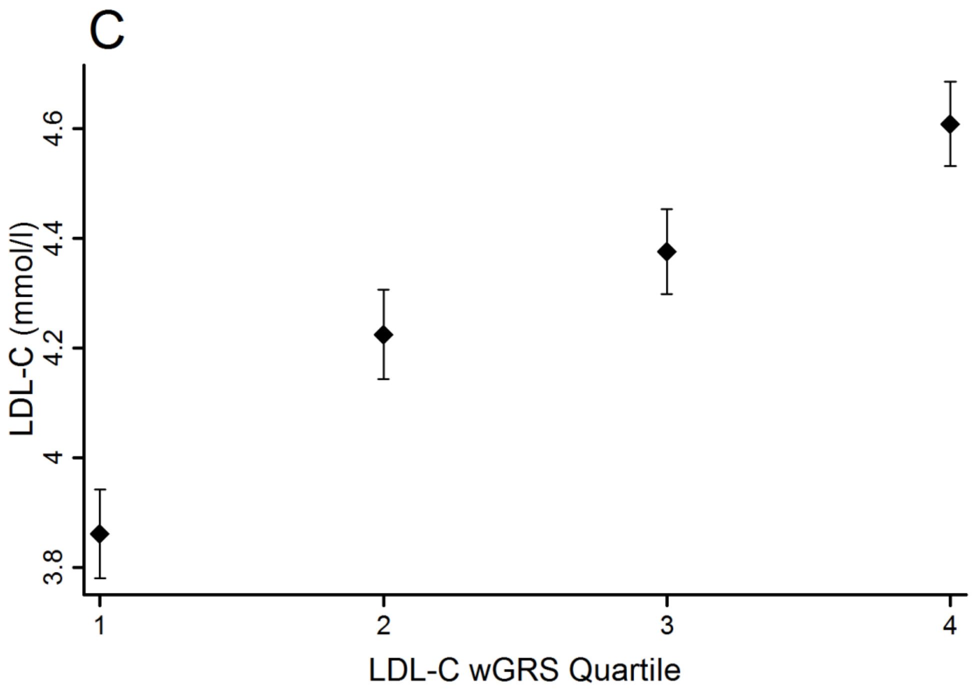
C
LDL-C (mmol/l)
3.8
4
4.2
4.4
4.6
1
2
3
4
LDL-C wGRS Quartile

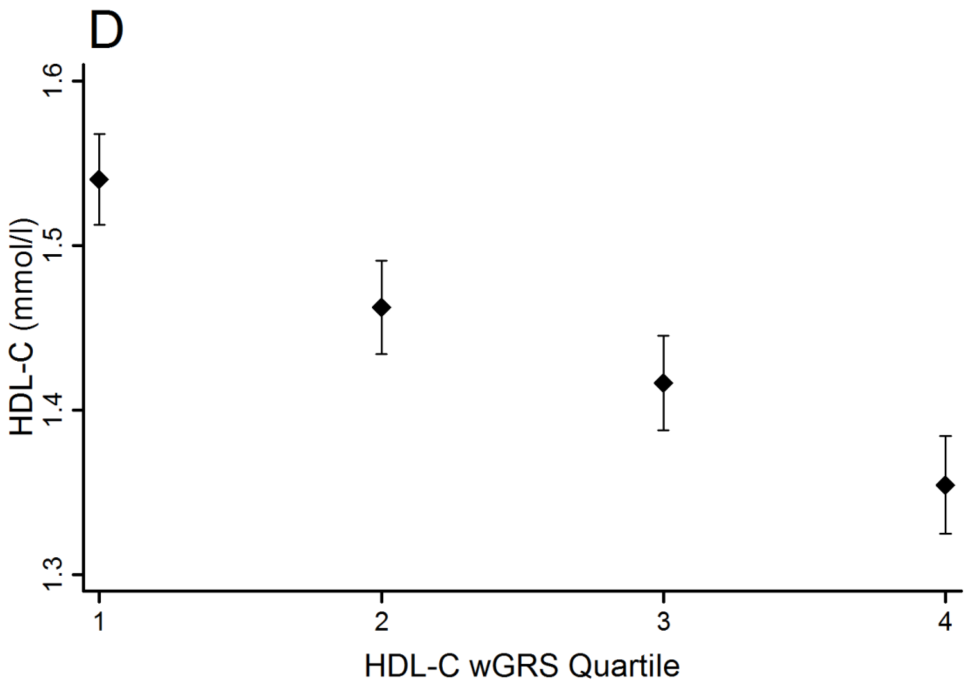
D
HDL-C (mmol/l)
1.3
1.4
1.5
1.6
1
2
3
4
HDL-C wGRS Quartile
